# Managing and treating COVID-19 in patients with hematological malignancies: a narrative review and expert insights

**DOI:** 10.1007/s10238-024-01381-5

**Published:** 2024-06-04

**Authors:** Heng Joo Ng, Maaz Kamal Alata, Quang The Nguyen, Phu Huynh Duc Vinh, Jing Yuan Tan, Chieh Lee Wong

**Affiliations:** 1 Department of Haematology, Singapore General Hospital, Singapore, Singapore; 2 Prince Mohammed Bin Nasser Hospital, Jazan, Saudi Arabia; 3 Stem Cell Transplantation Department, Blood Transfusion Hematology Hospital, Ho Chi Minh, Vietnam; 4 Department of Haematology, Sunway Medical Centre, Bandar Sunway, Selangor Malaysia; 5 School of Medical and Life Sciences, Sunway University, Bandar Sunway, Selangor Malaysia

**Keywords:** COVID-19, Omicron, Hematological malignancies, Immunocompromised, Immunosuppressed, Lymphomas

## Abstract

Patients with hematologic malignancies (HMs) are at a significantly higher risk of contracting COVID-19 and experiencing severe outcomes compared to individuals without HMs. This heightened risk is influenced by various factors, including the underlying malignancy, immunosuppressive treatments, and patient-related factors. Notably, immunosuppressive regimens commonly used for HM treatment can lead to the depletion of B cells and T cells, which is associated with increased COVID-19-related complications and mortality in these patients. As the pandemic transitions into an endemic state, it remains crucial to acknowledge and address the ongoing risk for individuals with HMs. In this review, we aim to summarize the current evidence to enhance our understanding of the impact of HMs on COVID-19 risks and outcomes, identify particularly vulnerable individuals, and emphasize the need for specialized clinical attention and management. Furthermore, the impaired immune response to COVID-19 vaccination observed in these patients underscores the importance of implementing additional mitigation strategies. This may include targeted prophylaxis and treatment with antivirals and monoclonal antibodies as indicated. To provide practical guidance and considerations, we present two illustrative cases to highlight the real-life challenges faced by physicians caring for patients with HMs, emphasizing the need for individualized management based on disease severity, type, and the unique circumstances of each patient.

## Introduction

The COVID-19 pandemic, caused by the SARS-CoV-2 virus, has significantly impacted public health worldwide. Infection rates, morbidity, and mortality have varied across geographic regions and time periods, characterized by successive waves of infections driven by emerging variants [1–6]. Data indicate a general decrease in mortality and disease severity with each wave, except for the Delta variant-dominant wave, which had higher mortality rates [1, 3, 7, 8]. On the other hand, the Omicron wave has been associated with less severity and mortality, potentially due to the variant itself or to acquired immunity from prior infection or vaccination, both of which have shown to substantially decrease the risk of severe infection in later waves [8].

Certain risk factors consistently linked to infection severity or mortality includes advanced age, male sex, and the presence of comorbidities [3, 4, 9]. A particularly vulnerable group includes individuals with HMs, which encompass cancers of the blood including leukemias, lymphomas, and myelomas [10]. As a group, HMs are the fourth most frequently diagnosed type of cancer in both men and women [11], affecting approximately 1.3 million individuals worldwide [12]. Impaired immune regulation in HM patients, compounded by immunosuppressive treatments, places them at increased risk for life-threatening infection [13].

Although the world has transitioned to an endemic state, clinicians should remain vigilant when managing HM patients, and understanding the ongoing implications and risks associated with COVID-19 remains as important as ever. Studies reported that while COVID-19 infection rates in HM patients are comparable to the general population, significant differences exist in morbidity and mortality [14–16]. Notably, elderly individuals with HMs are particularly vulnerable due to their advanced age and increased risk of contracting nosocomial infections from assisted living or healthcare facilities [17].

This paper offers a comprehensive review of the impact of COVID-19 on individuals with HMs utilizing a combination of literature analysis and expert experience. We examine key clinical aspects, including the risks of severe COVID-19 influenced by different HM subtypes and clinical factors, as well as the impact of cancer treatments on COVID-19 outcomes and vaccine-induced seroconversion and protection levels. Considering the rapidly evolving disease landscape along with the accumulation of experience and knowledge, we also discuss the various prophylactic and therapeutic options for the clinical management of HM patients. We presented two hypothetical patient scenarios that shed light on the complexities and considerations involved in the care of HM patients. Drawing on real-life insights from an international group of experts, our manuscript aims to provide a highly practical review, offering insights and best practices for effectively addressing the ongoing threat of COVID-19 among individuals with HM.

## Understanding the impact of HM on COVID-19-related risks

Studies consistently indicate that individuals with HMs are at higher risks of severe COVID-19 infection, secondary infections, and hospitalizations compared to the general population [18, 19]. Moreover, compared to individuals with solid tumors, those with HMs had a greater susceptibility to severe or moderate COVID-19 [20], possibly due to the depletion of antibodies and antibody-producing B cells associated with HMs [21].

A meta-analysis reported an approximate 25% mortality rate among HM patients hospitalized for COVID-19 [22]. Several factors contribute to the elevated all-cause mortality in this group, including direct infection, lymphopenia, interruption of cancer treatment, immunosuppression due to treatments, and secondary infections. While the general population experienced decreased COVID-19 mortality rates from the initial phase to the phase dominated by the Omicron variant [1], this decline was not initially observed in individuals with HMs until widespread vaccination [23–25]. Throughout the pandemic, HM patients consistently exhibited higher mortality rates compared to both the general population and individuals with solid tumors [20–22].

### COVID-19-associated thrombosis and complications in HMs

Patients with HMs and COVID-19 have higher morbidity and mortality; however, the underlying pathophysiological mechanisms remain unclear. It has been postulated that endothelial injury plays a central role in the pathogenesis of acute respiratory distress syndrome and organ failure in severe COVID-19 [26]. Moreover, COVID-19 is associated with complex coagulation abnormalities, leading to a hypercoagulable state and thrombosis [26, 27]. Stasis, common in hospitalized or critically ill patients, irrespective of COVID-19, is a well-known contributor to the development of venous thromboembolism (VTE) [27]. Patients with COVID-19 exhibit various prothrombotic factors, including elevated factor VIII, as well as laboratory abnormalities indicating increased fibrinogen and D-dimer levels [27]. This phenotypic hypercoagulable state has been referred to as "COVID-19-associated coagulopathy or thromboinflammation" [28]. In parallel, cancer itself is associated with a hypercoagulable state and a significantly higher incidence of thromboembolic complications [26, 27]. Therefore, the combination of HM and COVID-19 may amplify this risk, resulting in overall poor outcomes [26, 27].

A retrospective study of HM patients reported statistically significant higher rates of composite thrombotic outcomes (cerebrovascular accidents + VTE) compared to the general population [27]. Independent of disease status, HM patients also exhibited a significantly increased need for intensive care and respiratory support, and had higher fatality rates [27]. Based on these findings, it is recommended that patients with HM be treated with anticoagulation strategies to mitigate the risk of thrombotic complications and optimize patient outcomes. The latest American Society of Hematology guidelines recommend prophylactic-dose anticoagulants for COVID-19 patients needing ICU care in the absence of contraindications, over intermediate or therapeutic doses, as VTE prophylaxis, consistent with other international recommendations from the US National Institutes of Health COVID-19 Treatment Guideline, the World Health Organization Living Guidance document, and the International Society on Thrombosis and Haemostasis [29].

In the following, we present an illustrative case scenario that exemplifies the increased risk of severe COVID-19 and the associated complications faced by a HM patient undergoing chemotherapy.

### Illustrative cases

*The case scenarios presented are for illustrative purposes only and do not represent specific individuals. They are hypothetical scenarios derived from the authors’ collective clinical experience.

## Scenario 1–An elderly patient with marginal zone lymphoma

### Background

A 70-year-old male was diagnosed with Stage 4E marginal zone B cell lymphoma involving the kidney and bone marrow. He completed guideline-recommended treatment with 6 courses of the rituximab-bendamustine combination in October 2021, resulting in complete remission. Subsequently, he received rituximab maintenance therapy every 8 weeks, completing his second cycle in March 2022. He had received 3 doses of COVID-19 mRNA vaccine.

### COVID-19 clinical management

**Fig. 1:**
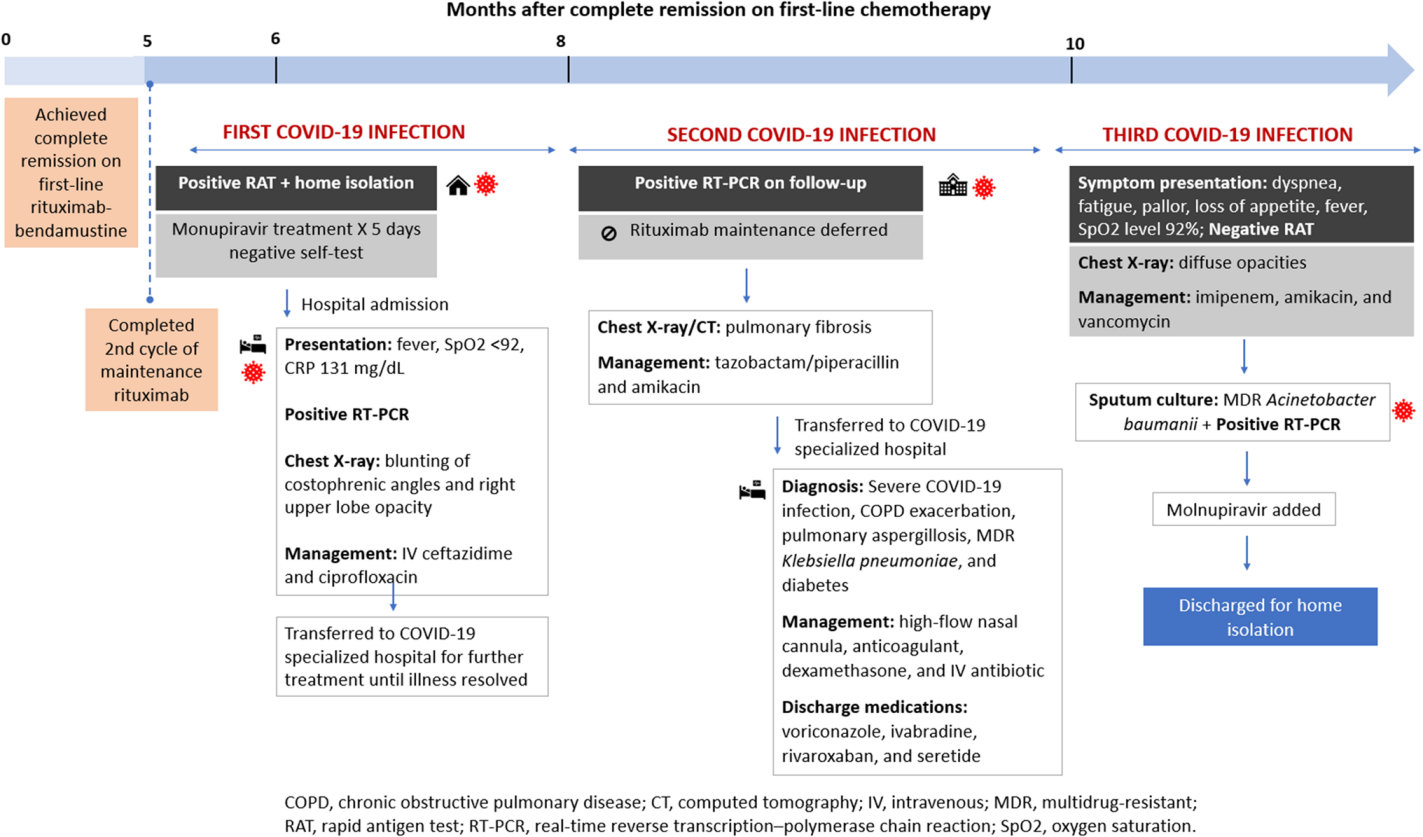
Summary of COVID-19 clinical history/timeline

The patient experienced three separate COVID-19 infections, as described in Fig. 1. The first time, he received treatment with molnupiravir and recovered. However, he was subsequently tested positive for COVID-19 on follow-up and developed a fever. As a result, the planned third course of rituximab maintenance therapy was deferred. The patient was transferred to a COVID-19-specialized hospital for further treatment. There, he was treated for severe COVID-19 infection, pulmonary aspergillosis, and multidrug-resistant Klebsiella pneumoniae. Shortly after discharge, the patient returned to the clinic with episodes of dyspnea and fever and tested positive for COVID-19 once again. The patient was treated with additional medications and requested home isolation.

### Resolution of COVID-19 and current status

On follow-up assessments, the patient was clinically stable but had shortness of breath with activities and required considerable assistance. His marginal zone lymphoma remained in remission. Chest CT showed worsened lung fibrosis and ground-glass opacities.

Currently, the patient is considerably stable, with weight gain, and has no more shortness of breath. Rituximab maintenance therapy was discontinued to prevent further immunosuppression.

### Summary

This case highlights the heightened vulnerability of a patient with HM to severe COVID-19 disease, further compounded by age, as evidenced by multiple recurrent infections and failure of virus clearance. The patient experienced severe secondary conditions, including pneumonia, resulting in long-term lung damage. The case emphasizes that the severity of COVID-19 extends beyond the acute phase, as immunocompromised individuals remain at risk even after completing chemotherapy and transitioning to maintenance therapy. Therefore, careful outpatient monitoring and up-to-date vaccinations are crucial. Considering the patient's susceptibility to infections and ongoing cancer remission, the decision was made to withhold rituximab maintenance to prevent further immunosuppression.

## Impact of COVID-19 on different types of HM

**Fig. 2:**
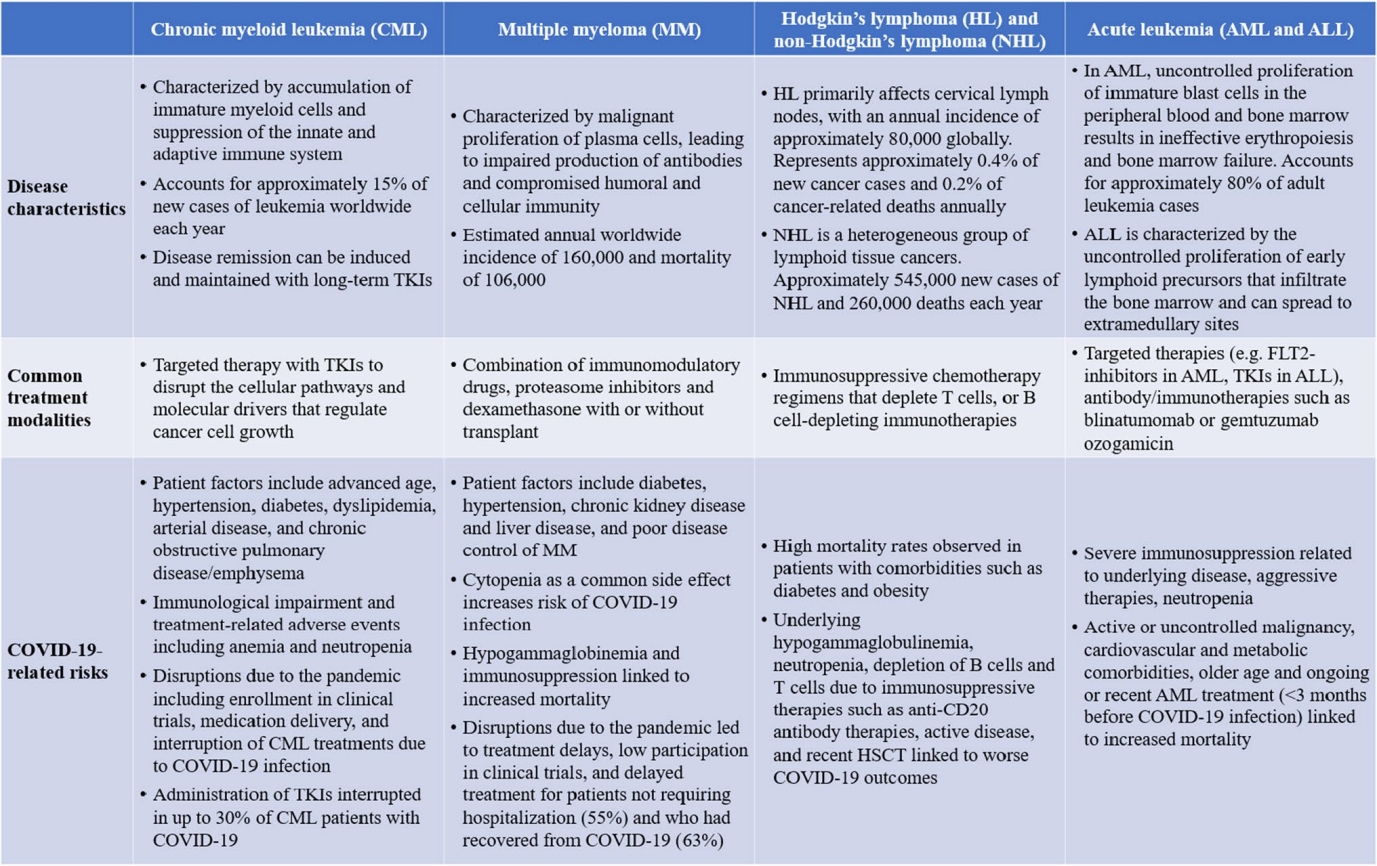
Summary overview of HM disease characteristics, common treatment modalities, and COVID-19-related risks [30–51]

The impact of COVID-19 on individuals with HMs differs from those without HMs and even individuals with solid tumors. Further examination focusing on different types of HMs (Fig. 2) is necessary to understand the potential varying effects of COVID-19 on these patients and to develop appropriate strategies for managing and preventing adverse outcomes.

### Chronic myeloid leukemia (CML)

CML patients typically require lifelong therapy with tyrosine kinase inhibitors (TKIs), which is linked to suppression of the innate and adaptive immune system [30]. Immune dysfunction is particularly pronounced at diagnosis [30]. The impact of targeted cancer treatments like TKIs on vaccination response and inhibition of B cell function and antibody response is also well documented [41].

Surprisingly, the incidence of COVID-19 infection in individuals with CML was relatively low, ranging from 4.1 to 6.7% across studies [52, 53]. Reported COVID-19 mortality varied, with some studies indicating lower rates compared to the general population and others reporting higher [42, 52, 53]. Data from earlier in the pandemic showed that active treatment with TKIs, particularly imatinib, was linked to reduced COVID-19 incidence and mortality, implying a potential protective effect [43].

The decreased incidence of COVID-19 in individuals with CML receiving active TKI treatment may be attributed to the resolution of immune deficiencies caused by the malignancy, which occurs within a few weeks of initiating treatment [33]. Further investigations suggest that TKIs may also play a role in reducing the impact of COVID-19 by inhibiting viral fusion to host cells [54], as previously demonstrated against other coronaviruses such as SARS-CoV and MERS-CoV [55, 56]. Additionally, TKIs have been found to upregulate key antiviral genes and downregulate proviral genes associated with the immune system [54], supporting their potential role in combating COVID-19 infection beyond their anticancer effects in CML patients. Therefore, despite their partly immunosuppressive function [41], TKIs appear to have a protective role against COVID-19, suggesting that TKI treatment should not be interrupted for CML patients with COVID-19 [33].

### Multiple myeloma (MM)

In MM, impaired antibody production and compromised immunity, coupled with treatment-related side effects such as cytopenia, increase susceptibility to respiratory infections like COVID-19 [57–59].

Larger evaluations have consistently shown higher rates of COVID-19 infection, hospitalization, and mortality in MM patients compared to those without cancer [36]. In contrast to the general population, COVID-19 survival rates in MM patients did not improve between the first and second waves [36], and the high COVID-19 mortality rate (ranging from 24 to 55%) [60] has been linked to hypogammaglobinemia and immunosuppression [57, 61].

The impact of anticancer treatments on COVID-19 outcomes in MM patients has been inconclusive. While some studies showed no negative outcomes and robust immune responses in MM patients receiving anticancer treatments [58, 61, 62], others indicated a higher risk of severe COVID-19 symptoms with active MM therapy [63, 64]. Findings on the anti-CD38 antibody daratumumab in relation to COVID-19 severity and complications have been mixed [61, 64]. Proteasome inhibitors and corticosteroids have been associated with an increased risk of severe COVID-19 outcomes, including ICU admissions and mechanical ventilation [45, 65, 66]. On the other hand, stem cell transplant within a year of infection does not appear to increase the risk of severe COVID-19 outcomes and, in some cases, shows more favorable outcomes [36, 45, 58, 67, 68].

### Hodgkin’s lymphoma (HL) and non-Hodgkin’s lymphoma (NHL)

Lymphomas, characterized by hypogammaglobulinemia, neutropenia, and depletion of B cells and T cells, place patients at heightened risk of infections like COVID-19 [39, 40].

Individuals with NHL have shown a higher COVID-19 incidence compared to other HMs [15, 69, 70]. Particularly, NHL patients requiring hospitalization were more likely to experience severe respiratory deterioration [70]. In an Italian cohort study, lymphoma patients had a high fatality rate of 33% due to COVID-19 [40]. Among different lymphoma subtypes, HL patients exhibited more favorable survival rates, primarily attributed to their significantly younger age, as supported by existing literature. Additionally, patients who received an initial lymphoma diagnosis 3 or more years before COVID-19 infection experienced better clinical outcomes and lower fatality, providing valuable insights for identifying patients for early vaccination strategies [40].

Active disease at the time of COVID-19 infection or progressive lymphoma status is the strongest predictors of death [39]. Treatment with anti-CD20 immunotherapy within 12 months of COVID-19 diagnosis has been associated with poorer outcomes, including longer hospitalization and increased mortality compared to individuals not receiving B cell-depleting therapies [71]. Lymphoma patients who have undergone hematopoietic stem cell transplantation (HSCT) also showed prolonged COVID-19 infection [71].

Considering these findings, careful monitoring and longer-term clinical follow-up are warranted to assess the impact of lymphoma and its treatment on immunity and COVID-19 outcomes. Patients should receive optimal treatment for their underlying disease, with a focus on achieving disease remission to improve outcomes [39].

### Acute leukemias (AML and ALL)

To date, there is limited data on COVID-19 in adult patients with acute leukemia (AL) particularly in relation to acute myeloid leukemia (AML) and (ALL). Effective management of patients with AL and concurrent COVID-19 necessitates close interdisciplinary collaboration between hematologists and infectiologists. On one hand, newly diagnosed AL requires prompt initiation of chemotherapy [50]. On the other hand, intensive therapy may increase the risk of severe COVID-19 [50]. Therefore, careful consideration is needed when determining the systemic treatment approach.

Pre-vaccination and pre-Omicron era data from the European Hematology Association Survey indicate a high COVID-19-related mortality rate of 40% in adult AML and 26% in adult ALL patients [72]. Other registry studies also support the elevated COVID-19 mortality observed in AML patients [50]. However, large data sets are lacking for adult ALL patients with COVID-19 due to the low ALL incidence in adults [50]. The impact of COVID-19 infections on AL treatment is rarely reported; however, available evidence suggests that treatment delay did not increase the risk of relapse, whereas therapy discontinuation was linked to worse outcomes in AML patients [50, 73]. Therefore, it is recommended to delay systemic treatment in AL patients with COVID-19 until SARS-CoV-2 negativity, unless immediate treatment is required [50]. These patients should receive early antiviral therapy to prevent disease progression and enable rapid elimination of the virus [50].

## Clinical factors linked to COVID-19 outcomes in HMs

### Types of immunotherapies

Since the start of the pandemic, the effects of immunosuppression resulting from HM treatments on COVID-19 outcomes have been a concern. Emerging evidence indicates that the effects of immunosuppressive treatments vary. Although certain HM treatments are known to induce neutropenia, it has been suggested that neutropenia may not be entirely detrimental in COVID-19 cases, as neutrophils are likely mediators of COVID-19-related pulmonary damage [74]. Additionally, certain immunosuppressants, like TKIs, may offer some potential benefits through antiviral activity [43, 54]. On the other hand, B cell-suppressing immunotherapies [71], chemotherapy regimens like platinum plus etoposide, or DNA methyltransferase inhibitors, have been shown to negatively impact COVID-19 outcomes [75].

Multiple studies indicated worse outcomes for HM patients on immune-suppressing chemotherapy in the 1–3 months prior to contracting COVID-19, including higher hospitalization, ICU admissions, mechanical ventilation, and death [22, 24, 76]. However, the impact of recent systemic anticancer therapies on COVID-19 outcomes is complex, with mixed findings [77, 78]. Some studies suggested increased mortality risk with systemic conventional chemotherapy, but not with monoclonal antibody or molecular-targeted therapies [24]. Specific regimens, including R-CHOP (rituximab, cyclophosphamide, doxorubicin, vincristine, and prednisolone), platinum plus etoposide, and DNA methyltransferase inhibitors, were associated with particularly high all-cause mortality rates (> 40%) [75]. Additionally, significant mortality rates were observed in HM patients receiving systemic corticosteroid therapy.

Altogether, these findings underscore the importance of considering specific COVID-19 prevention measures and carefully managing treatment regimens. While discontinuing anticancer treatment can lead to worsened outcomes, certain therapies carry a heightened risk of COVID-19 mortality.

### Immunologic factors related to COVID-19-related mortality in HMs

Immunologic factors related to COVID-19 outcomes and mortality have attracted significant research interest. Lymphocytes, particularly CD8+ T cells, are known to play a crucial role in COVID-19 recovery [21, 79, 80]. Lymphopenia is common in patients with severe COVID-19 and is a known predictive factory for mortality [81]. In patients with lymphoid malignancies, impaired CD8+ T cell immunity, driven by B cell depletion, was associated with persistent COVID-19 infection, and individuals with low T cell counts (CD8+  < 50 cells/μL or CD4+  < 100 cells/μL) had markedly poorer outcomes, including higher 60-day mortality rates [79]. Severe lymphopenia was also associated with higher risks of secondary infections within 48 h after hospital admission, contributing to increased mortality [81]. Among HM patients, those who developed secondary infections had a markedly higher 30-day mortality rate compared to those without secondary infections (69% vs. 15%, *p* < 0.001) [82].

HM patients are particularly vulnerable due to immune cell depletion, including CD8+ T cells and B cells, caused by underlying malignancies and immunosuppressive therapies [21]. Moreover, disruptions to cancer treatments during the pandemic, either as a protective measure or due to COVID-19 complications, are associated with decreased survival post-infection [83, 84].

In a prospective cohort study, patients with solid tumors exhibited similar immunologic profiles to patients without cancer during acute COVID-19, while patients with HM showed significant impairments in B cell function and SARS-CoV-2-specific antibody responses [21]. Interestingly, despite the impaired humoral immunity and higher mortality in HM patients with COVID-19, improved survival rates were observed in those with higher CD8+ cells, including individuals on anti-CD20 therapy [21]. Notably, 77% of HM patients demonstrated detectable SARS-CoV-2-specific T cell responses, underscoring the crucial role of cellular immunity when humoral responses are compromised [21]. This finding is promising because current COVID-19 mRNA vaccines induce robust CD8+ T cell responses alongside humoral responses [21]. It is important to note that both B cell-depleting therapies and cytotoxic chemotherapy agents, which can impact the T cell compartment, are mainstays of treatment in lymphoma [21]. Therefore, treatment decisions should carefully weigh the risk of immune dysregulation against the benefits of disease control, particularly in non-curative settings.

### Impact of B cell versus T cell depletion

A study comparing COVID-19 outcomes in individuals with HMs who had prolonged infection found that T cell-depleting therapies were associated with increased COVID-19 mortality, while B cell-depleting therapies were linked to rehospitalization and prolonged COVID-19 infections [79]. The complex interaction between B cells and T cells may explain these findings, as B cells rely on CD4+ T cells for maturation [79].

B cells are crucial for immune defense against viral infections, producing viral-specific antibodies and triggering cytokine responses important for clearing infections like SARS-CoV-2 [85]. Secondary immunodeficiency commonly occurs in HMs and often affects B cells [86]. In advanced HMs such as CLL, MM, and certain forms of NHL, dysregulation of immune cells results in B and T cell depletion and hypogammaglobulinemia. Treatments for these HMs, including monoclonal antibodies targeting B cell epitopes, have been linked to poorer COVID-19 outcomes [86]. Particularly, evidence consistently demonstrates the impact of anti-CD20 agents, such as rituximab, in inducing rapid B cell depletion, resulting in prolonged COVID-19 courses characterized by persistent SARS-CoV-2 replication [71]. Blinatumomab, a bispecific CD19-directed T cell engager, is used for treatment of ALL can also impair B cell response and causes hypogammaglobulinemia [87]. There are currently no specific recommendations regarding SARS-CoV-2 and blinatumomab, so the decision to withhold therapy should consider its risks and benefits.

## Scenario 2–A patient with B-ALL on blinatumomab

### Background

A 55-year-old female with no significant past medical history was diagnosed with B cell ALL in August 2022. She received one cycle of intensive induction chemotherapy which was complicated by severe fungal infection, precluding her from further intensive chemotherapy. She was subsequently switched to blinatumomab for further treatment of her B-ALL.

### COVID-19 clinical management

In May 2023, she tested positive for COVID-19 during her second cycle of blinatumomab infusion. She presented with fever, cough, and rhinorrhea but did not require oxygen therapy. Chest X-ray was clear. She had previously received three doses of mRNA vaccine (last dose in May 2022). She was admitted for monitoring and received a 5-day course of intravenous remdesivir with improvement of her symptoms. She was discharged on day 5 of hospital admission with no major complications. However, her COVID-19 took up to 5 weeks to fully resolve, as indicated by persistent weekly positive results on nasopharyngeal COVID-19 PCR swabs. As a result, her cancer treatment had to be delayed.

### Summary

This case underscores the challenges faced by patients undergoing immunosuppressive cancer treatment, including the increased risk of severe outcomes, such as hospitalization and susceptibility to secondary infections, due to both the treatment itself and inadequate protection from COVID-19 vaccinations. Despite completing the full course of COVID-19 vaccination, they may still face an elevated risk of persistent COVID-19, with prolonged symptoms and positive PCR tests, likely due to impaired immune responses. These complications not only prolong the illness but also result in delays in HM maintenance treatment.

## COVID-19 management in individuals with HMs

### Vaccination

**Fig. 3:**
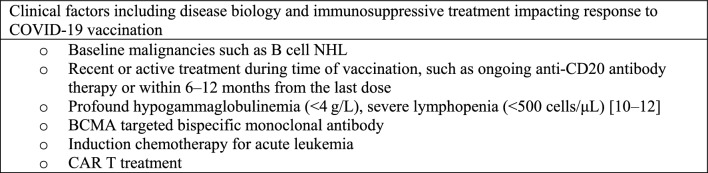
Factors identified to be associated with poor COVID-19 vaccine response [89–93]

Vaccination is a significant component in the COVID-19 armamentarium, with proven efficacy in reducing transmission, hospitalizations, and deaths due to severe COVID-19 in the general population. However, concerns have been raised regarding its effectiveness in individuals with HMs, as well as waning immunity over time [88, 89]. While multiple vaccines have been developed, studies primarily focus on mRNA-based vaccine use in immunosuppressed and HM patients [88]. Initial reports have indicated lower seroconversion in individuals with HMs following COVID-19 vaccination, attributed to impaired humoral immunity and the effects of immunosuppressive therapies (Fig. 3) [88, 89].

A systematic review found seroconversion rates ranging from 54 to 85% in HM patients after receiving two vaccine doses, compared to nearly 100% in healthy controls or from 85 to 94% in individuals with solid tumors [94]. Notably, those receiving B cell-depleting therapies and recent immunosuppressive treatments, including hematopoietic stem cell transplantation (HSCT), had significantly lower seroconversion rates [88, 94]. Similarly, lower seroconversion rates were observed with anti-CD20 antibodies [83, 94–96], Bruton’s tyrosine kinase inhibitors (BTKIs) [83], and stem cell transplantation [95]. Earlier studies on chimeric antigen receptor T cell (CAR-T) therapy and bispecific antibodies have demonstrated variable SARS-CoV-2-specific B and T cell responses in patients with MM [97, 98]. Particularly, patients undergoing active treatment with anti-CD38 and anti-B cell maturation antigen (BCMA) antibody-based therapies showed an unexpected absence of T cell responses and anti-spike antibodies post-vaccination compared to those not on active treatment [97, 98]. Additional studies confirmed that only a minority (~ 30%) of MM and NHL patients receiving CAR-T had clinically relevant antibody responses (> 250 IU/mL) [99]. On the other hand, immune checkpoint inhibitor therapies or hormonal therapies did not appear to impact vaccine efficacy, as individuals with HMs receiving these treatments demonstrated seroconversion rates of 97% and 100%, respectively [95].

Despite the lower seroconversion rates observed in HM patients, substantial evidence supports the clinical benefit of COVID-19 vaccination, with emerging data indicating a dose–response relationship [89, 100]. A higher number of vaccine doses received before COVID-19 infection is associated with significantly lower mortality rates, highlighting the benefit of booster doses in providing optimal protection to this vulnerable group [89]. CAR-T recipients showed improved serological response rates with each additional vaccine dose, reaching 75% after four doses compared to 20.4% after one dose [9]. Furthermore, anti-spike antibody titers were more than 30 times higher after four doses compared to two doses [100]. Based on emerging data and the evolving context of COVID-19, revised vaccination recommendations for patients with HM and HSCT (Table 1) now include a three-dose primary schedule followed by an additional vaccine dose. [90] The administration of a fourth vaccine dose has been shown to be safe and effective in increasing antibody titers [90]. It is crucial to prioritize timely vaccination without delaying the treatment of underlying diseases, and even patients with expected poor response, such as those on therapy with anti-CD20 antibodies, may still benefit from vaccination [90].

**Table 1:** Updated recommendations of ECIL 9 (European Conference on Infections in Leukemia) on vaccinations for COVID-19 in HM and HSCT patients [90]

Key highlights of vaccination recommendations for HM patients including HSCT and CAR-T cell recipients
A three-dose primary schedule of mRNA vaccine is recommended; additional booster dose(s) or mRNA vaccine should be considered after at least 3 months from the 3rd dose
The interval between a COVID-19 infection and subsequent boosters should be at least 3, and preferably 4 months
For HSCT recipients, additional doses can help improve immune response by increasing seroconversion and antibody levels. Therefore, booster doses are encouraged, preferably with the new updated bivalent vaccines targeting original strains and new Omicron subvariants. The risk of worsening/eliciting graft-versus-host disease should be considered when planning vaccination schedule
For CAR-T cell recipients, patients with B cell aplasia are unlikely to mount an antibody response; however, repeated vaccine doses might provide some benefit

#### Prophylactic measures

To enhance COVID-19 prevention in HM patients, it is crucial to uphold strict infection control measures, including hand hygiene, physical distancing, and ventilation of room [90]. The ECIL 9 guidelines highlight that it is essential for health personnel to utilize personal protective equipment, and patients should be placed in single rooms, avoiding positive pressure rooms, to effectively prevent transmission [88, 90].

Given the unpredictable or inadequate immune response to COVID-19 vaccination in HM patients, passive immunization strategies via pre- or post-exposure prophylaxis have shown to be valuable options for providing added protection to this vulnerable population [101]. These strategies involve the use of various anti-spike monoclonal antibodies, such as tixagevimab-cilgavimab, casirivimab-imdevimab, and bamlanivimab-etesevimab, which work by binding to the SARS-CoV-2 spike protein and preventing viral entry into host cells [102].

In a large placebo-controlled randomized controlled trial, tixagevimab-cilgavimab administered as pre-exposure prophylaxis reduced the risk of developing symptomatic COVID-19 by 76.7% for a median of 83 days post-administration among high-risk patients with poor vaccine response [103]. The effectiveness of casirivimab-imdevimab as post-exposure prophylaxis was assessed in unvaccinated individuals who had been in contact with COVID-19 patients. Compared to placebo, casirivimab-imdevimab administered 96 h after diagnosis was associated with a lower risk of symptomatic COVID-19 (1.5% vs. 7.8%) [104].

However, it is important to note that studies on monoclonal anti-S antibodies as prophylactic strategies were mostly conducted before the predominance of the Omicron variant, particularly the newer BA.4/5 subvariants [101]. The neutralizing efficacy of casirivimab/imdevimab, bamlanivimab/etesevimab, and sotrovimab against BA.4/5 is significantly reduced to maintain clinical efficacy, while the efficacy of tixagevimab/cilgavimab is moderately reduced [101]. Preliminary data suggest doubling the dosage of tixagevimab/cilgavimab in the presence of less susceptible omicron subvariants may increase efficacy [105]. Considering these findings, it is important to evaluate each patient case’s individually, and pre-exposure prophylaxis should not be used as a substitute for vaccination when successful vaccination is feasible [90, 101]. Monoclonal anti-spike antibodies for pre- and post-exposure prophylaxis can be considered if they demonstrate activity against circulating variants [90].

#### Treatment

The treatment options for COVID-19 are constantly evolving. Several crucial factors need to be considered when determining treatment strategies for HM patients with COVID-19, including the patient’s immune status, prior COVID-19 vaccinations and anticipated vaccine response, severity of COVID-19, local epidemiology and presence of VOCs, as well as the availability of anti-COVID-19 drugs [101]. Treatment strategies are tailored based on the differentiation of disease severity and taking into account the scale of clinical progression, ranging from mild outpatient cases to moderate or severe hospitalized cases [90, 101]. Table 2 provides a summary of currently used therapies. The importance of early treatment initiation cannot be overstated.

**Table 2:** Summary of current treatment options for patients with COVID-19. List based on the latest ECIL 9 and Infectious Diseases Working Party (AGIHO) of the German Society for Hematology and Medical Oncology guidelines (at time of manuscript preparation) [90, 101]

	Mild COVID-19	Moderate to severe COVID-19 (requiring oxygen support; or saturation < 90–94%, respiratory rate ˃ 30/min)	Critical COVID-19 (ARDS, sepsis, septic shock, MIV, NIV, or vasopressor therapy)
Treatment options	a) Anti-SARS-CoV-2 monclonal antibodies, if active against the circulating variants b) Nirmatrelvir/ritonavir c) Remdesivir d) Molnupiravir -Dexamethasone is not recommended in the early treatment of mild/outpatient HM patients with COVID-19	a) Dexamethasone b) Remdesivir c) If patient is seronegative: monoclonal antibodies, if active against the circulating variants or high-titer convalescent plasma, if monoclonal antibodies are not accessible d) If severe COVID-19 inflammation (indicated by inflammation parameters), including worsening despite dexamethasone, add 2nd immunosuppressant: Anti-IL-6 (tocilizumab, sarilumab) or JAK inhibitor(baracitinib/tofacitinib) Anti-IL 1 (anakinra)	a) Dexamethasone b) Remdesivir c) Monoclonal antibodies, if active against the circulating variants (no data in MIV patients) d) If severe COVID-19 inflammation (indicated by inflammation parameters), add 2nd immunosupprssant with anti-IL-6 (tocilizumab, sarilumab)

ARDS, acute respiratory distress syndrome; MIV, mechanical invasive ventilations; NIV, non-invasive ventilation

##### Mild–moderate disease

For patients with mild-to-moderate disease, antivirals and monoclonal antibodies are effective options for early therapy [90, 101]. Randomized controlled trials have demonstrated the effectiveness of initiating these treatments within 3–7 days from symptom onset in reducing hospitalization or death in unvaccinated outpatients with mild or moderate COVID-19 or those at high risk for severe disease [90, 101]. Although HM patients constituted only a small minority in these trials, observational studies support the notion that this population can benefit from these treatments [90, 106]. Moreover, since most of these trials exclude vaccinated patients, the evidence can be best extrapolated to cancer patients who are either unvaccinated or expected to have an inadequate vaccine response [101].

Monoclonal antibodies or antibody combinations, including casirivimab/imdevimab, bamlanivimab/etesevimab, regdanvimab, sotrovimab, and tixagevimab/cilgavimab, have shown significant reductions in hospitalization or death (ranging from 50.5 to 85%) compared to placebo [101]. However, data on their efficacy against the Omicron variant, particularly the BA.4/5 subvariants, are primarily based on in vitro or small observational studies [101]. Therefore, antivirals remain the cornerstone of therapy as their activity is not influenced by VOCs [90]. Currently, three antiviral agents are used for early COVID-19 treatment in high-risk patients: nirmatrelvir/ritonavir, remdesivir, and molnupiravir [90, 101].

Oral nirmatrelvir/ritonavir or intravenous remdesivir are the preferred choices based on efficacy data [107, 108]. When considering nirmatrelvir/ritonavir, potential drug-drug interactions should be taken into account, and careful assessment or reduction of existing immunosuppression or targeted therapy can allow its use in most HM patients [90]. Molnupiravir use is limited by the lower efficacy in the randomized trial (relative risk reduction of 30% [109], compared to 87% of nirmatrelvir/ritonavir or remdesivir), and might not be available in some countries however offers advantages such as the absence of drug-drug interactions and the possibility of use in patients with renal failure (ClCr < 30 ml/min) [90]. It can be considered as early therapy for ambulatory patients when more potent therapeutic options are contraindicated or unavailable [90, 110].

The routine use of high-titer convalescent plasma is not supported for the treatment of mild/moderate COVID-19 [90]. However, considering its reduced susceptibility to protein-spike mutations that can lead to the loss of monoclonal antibody activity, convalescent plasma might be useful in immunocompromised patients in addition to antivirals, especially when monoclonal antibodies effective against the locally predominant variants or antivirals are not available [90].

##### Moderate–severe disease requiring hospitalization or oxygen support

COVID-19 progresses through different phases, with active viral replication as the primary factor in the earlier stages and hyperinflammation becoming more prominent in later, more severe disease [101]. For the management of hospitalized HM patients with COVID-19, it is important to distinguish between moderate disease (requiring no or low-flow oxygen) and severe disease (requiring high-flow oxygen, non-invasive ventilation, or mechanical ventilation) [101]. Remdesivir can be considered for patients with moderate COVID-19 or with severe COVID-19 not yet on mechanical ventilation for up to 10 days [101]. However, COVID-19 patients, and cancer patients in particular, often experience rapid deterioration, with escalation from low-flow oxygen to mechanical ventilation within 24 h in some cases [101]. Therefore, the patient's disease course should be taken into account, and remdesivir can be combined with other adjuncts such as IL-6 or JAK inhibitors if necessary [90, 110].

Immunosuppressive agents, particularly dexamethasone, are an important part of therapy in severely ill COVID-19 patients. The benefit of dexamethasone in improving clinical outcomes and reducing mortality was demonstrated in about a fifth of patients with low- or high-flow oxygen and about a third in mechanically ventilated patients [101]. In the presence of systemic inflammation (e.g., highly elevated C-reactive protein, respiratory worsening) the addition of anti-IL-6 monoclonal antibodies such as tocilizumab or sarilumab to dexamethasone can be considered for patients on oxygen support [88, 90, 101]. Alternatively, for patients with systemic inflammation based on elevated levels of soluble urokinase plasminogen activator receptor, anti-IL-1 monoclonal antibodies such as anakinra have shown benefit. Additionally, JAK inhibitors like baricitinib and tofacitinib have demonstrated survival benefits in addition to dexamethasone by controlling inflammation in patients on oxygen support [88, 101].

## Conclusion

COVID-19 has significantly impacted the health and treatment course of individuals worldwide. While progress has been made in understanding the disease and developing vaccines and treatments that led to improvements in morbidity and mortality, individuals with HMs remain vulnerable to COVID-19-related risks and poor outcomes. It is crucial to implement comprehensive measures, including prophylactic and therapeutic options, as well as updated vaccination strategies, to mitigate risks in this population. Ongoing research is necessary to stay ahead of the evolving viral landscape and emerging variants. Meanwhile, awareness of our current knowledge on how the immune system best combats COVID-19, and how current HM treatments impact immune function must be appreciated, and treatment strategies implemented accordingly to ensure optimal outcomes, not only in achieving disease remission but also in preventing COVID-19 infections.

## Data Availability

No datasets were generated or analyzed during the current study.

## References

[1] Suligowski R, Ciupa T. Five waves of the COVID-19 pandemic and green–blue spaces in urban and rural areas in Poland. Environ Res. 2023;216: 114662. 36374652 10.1016/j.envres.2022.114662 PMC9617687

[2] Martella M, Peano A, Politano G, Onorati R, Gianino MM. Paediatric hospitalizations over three waves of COVID-19 (february 2020 to may 2021) in Italy: determinants and rates. PeerJ. 2023;11: e15492. 37377787 10.7717/peerj.15492 PMC10292193

[3] Anscombe C, Lissauer S, Thole H, Rylance J, Dula D, Menyere M, et al. A comparison of four epidemic waves of COVID-19 in Malawi; an observational cohort study. BMC Infect Dis. 2023;23:79. 36750921 10.1186/s12879-022-07941-y PMC9902830

[4] Miyashita K, Hozumi H, Furuhashi K, Nakatani E, Inoue Y, Yasui H, et al. Changes in the characteristics and outcomes of COVID-19 patients from the early pandemic to the delta variant epidemic: a nationwide population-based study. Emerg Microbes & Infect. 2023;12:2155250. 36469641 10.1080/22221751.2022.2155250 PMC9788709

[5] Dhama K, Nainu F, Frediansyah A, Yatoo Mohd I, Mohapatra RK, Chakraborty S, et al. Global emerging Omicron variant of SARS-CoV-2: impacts, challenges and strategies. J Infect Public Health. 2023;16:4–14. 36446204 10.1016/j.jiph.2022.11.024 PMC9675435

[6] Hsiao Y-W, Bray DJ, Taddese T, Jiménez-Serratos G, Crain J. Structure adaptation in Omicron SARS-CoV-2/hACE2: biophysical origins of evolutionary driving forces. Biophys J. 2023;S0006–3495(23):00580–5. 10.1016/j.bpj.2023.09.003 PMC10624932 37717145

[7] Seyler L, Van Nedervelde E, De Cock D, Mann C, Pien K, Allard SD, et al. Surfing the waves: differences in hospitalised COVID-19 patients across 4 variant waves in a Belgian University Hospital. Viruses. 2023;15:618. 36992327 10.3390/v15030618 PMC10057609

[8] Tanaka H, Chubachi S, Asakura T, Namkoong H, Azekawa S, Otake S, et al. Characteristics and clinical effectiveness of COVID-19 vaccination in hospitalized patients in Omicron-dominated epidemic wave—a nationwide study in Japan. Int J Infect Dis. 2023;132:84–8. 37086866 10.1016/j.ijid.2023.04.399 PMC10121147

[9] Malaeb R, Haider A, Abdulateef M, Hameed M, Daniel U, Kabilwa G, et al. High mortality rates among COVID-19 intensive care patients in Iraq: insights from a retrospective cohort study at Médecins Sans Frontières supported hospital in Baghdad. Front Public Health. 2023;11:1185330. 37719728 10.3389/fpubh.2023.1185330 PMC10501727

[10] Division of Cancer Prevention and Control, Centers for Disease Control and Prevention. Hematologic Cancer Incidence, Survival, and Prevalence [Internet]. Centers for Disease Control and Prevention. 2023 [cited 2023 Nov 9]. Available from: https://www.cdc.gov/cancer/uscs/about/data-briefs/no30-hematologic-incidence-surv-prev.htm

[11] Smith A, Howell D, Patmore R, Jack A, Roman E. Incidence of haematological malignancy by sub-type: a report from the haematological malignancy research network. Br J Cancer. 2011;105:1684–92. 22045184 10.1038/bjc.2011.450 PMC3242607

[12] Zhang N, Wu J, Wang Q, Liang Y, Li X, Chen G, et al. Global burden of hematologic malignancies and evolution patterns over the past 30 years. Blood Cancer J. 2023;13:1–13. 37193689 10.1038/s41408-023-00853-3 PMC10188596

[13] Shah V, Ko T, Zuckerman Ko M, Vidler J, Sharif S, Mehra V, et al. Poor outcome and prolonged persistence of SARS-CoV-2 RNA in COVID-19 patients with haematological malignancies King’s College Hospital experience. Br J Haematol England. 2020;190(e279):82. 10.1111/bjh.16935 PMC7307054 32526039

[14] Zaki A, Soomar SM, Khan DH, Shaharyar Sheikh H, Iftikhar R, Mir A, et al. Outcomes of COVID-19 infection in patients with hematological malignancies: a multicenter analysis from Pakistan. PLoS ONE. 2022;17: e0267139. 35446898 10.1371/journal.pone.0267139 PMC9022796

[15] Wood WA, Neuberg DS, Thompson JC, Tallman MS, Sekeres MA, Sehn LH, et al. Outcomes of patients with hematologic malignancies and COVID-19: a report from the ASH research collaborative data hub. Blood Adv. 2020;4:5966–75. 33278301 10.1182/bloodadvances.2020003170 PMC7724912

[16] Manzano JM, Muthu M, Kheder E, Mohammed A, Halm J, Dickson K, et al. Hospitalization characteristics and outcomes of patients with cancer and COVID-19 at a comprehensive cancer center. Support Care Cancer. 2022;30:7783–8. 35705751 10.1007/s00520-022-07209-w PMC9200437

[17] Asai Y, Nomoto H, Hayakawa K, Matsunaga N, Tsuzuki S, Terada M, et al. Comorbidities as risk factors for severe disease in hospitalized elderly COVID-19 patients by different age-groups in Japan. Gerontology. 2022;68:1027–37. 34999588 10.1159/000521000 PMC8805047

[18] Liang W, Guan W, Chen R, Wang W, Li J, Xu K, et al. Cancer patients in SARS-CoV-2 infection: a nationwide analysis in China. Lancet Oncol. 2020;21:335–7. 32066541 10.1016/S1470-2045(20)30096-6 PMC7159000

[19] Gupta A, Gonzalez-Rojas Y, Juarez E, Crespo Casal M, Moya J, Rodrigues Falci D, et al. Effect of sotrovimab on hospitalization or death among high-risk patients with mild to moderate COVID-19: a randomized clinical trial. JAMA. 2022;327:1236–46. 35285853 10.1001/jama.2022.2832 PMC8922199

[20] Castelo-Branco L, Tsourti Z, Gennatas S, Rogado J, Sekacheva M, Viñal D, et al. COVID-19 in patients with cancer: first report of the ESMO international, registry-based, cohort study (ESMO-CoCARE). ESMO Open. 2022;7: 100499. 35644101 10.1016/j.esmoop.2022.100499 PMC9080222

[21] Bange EM, Han NA, Wileyto P, Kim JY, Gouma S, Robinson J, et al. CD8+ T cells contribute to survival in patients with COVID-19 and hematologic cancer. Nat Med. 2021;27:1280–9. 34017137 10.1038/s41591-021-01386-7 PMC8291091

[22] Naimi A, Yashmi I, Jebeleh R, Imani Mofrad M, Azimian Abhar S, Jannesar Y, et al. Comorbidities and mortality rate in COVID-19 patients with hematological malignancies: a systematic review and meta-analysis. J Clin Lab Anal. 2022;36: e24387. 35385130 10.1002/jcla.24387 PMC9102765

[23] Pinato DJ, Aguilar-Company J, Ferrante D, Hanbury G, Bower M, Salazar R, et al. Outcomes of the SARS-CoV-2 omicron (B.1.1.529) variant outbreak among vaccinated and unvaccinated patients with cancer in Europe: results from the retrospective, multicentre on COVID registry study. Lancet Oncol. 2022;23:865–75. 35660139 10.1016/S1470-2045(22)00273-X PMC9162476

[24] Martínez-López J, De La Cruz J, Gil-Manso R, Alegre A, Ortiz J, Llamas P, et al. COVID-19 severity and survival over time in patients with hematologic malignancies: a population-based registry study. Cancers. 2023;15:1497. 36900296 10.3390/cancers15051497 PMC10001264

[25] Pagano L, Salmanton-García J, Marchesi F, López-García A, Lamure S, Itri F, et al. COVID-19 in vaccinated adult patients with hematological malignancies: preliminary results from EPICOVIDEHA. Blood. 2022;139:1588–92. 34748627 10.1182/blood.2021014124 PMC8577877

[26] Levi M, van Es N. COVID-19 associated coagulopathy and thrombosis in cancer. Thromb Res. 2022;213:S72–6. 36210564 10.1016/j.thromres.2021.12.006 PMC9134033

[27] Cook MR, Dykes K, White K, Desale S, Agrawal R, Fernandez S, et al. Thrombotic and clinical outcomes in patients with hematologic malignancy and COVID-19. Clin Lymphoma Myeloma Leuk. 2022;22:e452–8. 35058217 10.1016/j.clml.2021.12.011 PMC8710237

[28] Connors JM, Levy JH. Thromboinflammation and the hypercoagulability of COVID-19. J Thromb Haemost. 2020;18:1559. 32302453 10.1111/jth.14849 PMC9770920

[29] Cuker A, Tseng EK, Schünemann HJ, Angchaisuksiri P, Blair C, Dane K, et al. American Society of Hematology living guidelines on the use of anticoagulation for thromboprophylaxis for patients with COVID-19: march 2022 update on the use of anticoagulation in critically ill patients. Blood Adv. 2022;6:4975–82. 35748885 10.1182/bloodadvances.2022007940 PMC9236618

[30] Hughes A, Yong ASM. Immune effector recovery in chronic myeloid leukemia and treatment-free remission. Front Immunol. 2017;8:469. 28484463 10.3389/fimmu.2017.00469 PMC5402174

[31] Hu Y, Li Q, Hou M, Peng J, Yang X, Xu S. Magnitude and temporal trend of the chronic myeloid leukemia: on the basis of the global burden of disease study. JCO Glob Oncol. 2019;2021:1429–41. 10.1200/GO.21.00194 PMC8492379 34591599

[32] Atallah E, Sweet K. Treatment-free remission: the new goal in cml therapy. Curr Hematol Malig Rep. 2021;16:433–9. 34618317 10.1007/s11899-021-00653-1 PMC8495665

[33] Delgado N, Torres A. What do we currently know about chronic myeloid leukemia (CML) and COVID-19? Curr Oncol Rep. 2022;24:645–50. 35218499 10.1007/s11912-021-01169-w PMC8881701

[34] Ludwig H, Novis Durie S, Meckl A, Hinke A, Durie B. Multiple myeloma incidence and mortality around the globe; interrelations between health access and quality, economic resources, and patient empowerment. Oncologist. 2020;25:e1406–13. 32335971 10.1634/theoncologist.2020-0141 PMC7485361

[35] Zhou L, Yu Q, Wei G, Wang L, Huang Y, Hu K, et al. Measuring the global, regional, and national burden of multiple myeloma from 1990 to 2019. BMC Cancer. 2021;21:606. 34034700 10.1186/s12885-021-08280-y PMC8152089

[36] Martinez-Lopez J, Hernandez-Ibarburu G, Alonso R, Sanchez-Pina JM, Zamanillo I, Lopez-Muñoz N, et al. Impact of COVID-19 in patients with multiple myeloma based on a global data network. Blood Cancer J. 2021;11:198. 34893583 10.1038/s41408-021-00588-z PMC8661359

[37] Hjalgrim H, Jarrett RF. Epidemiology of hodgkin lymphoma. In: Engert A, Younes A, editors. Hodgkin lymphoma a comprehensive overview. Cham: Springer International Publishing; 2020. p. 3–23.

[38] Huang J, Rohatgi A, Schneider J, Braunstein M. Considerations for the management of oncology patients during the COVID-19 pandemic. Oncology (Williston Park). 2020;34:432–41. 33058111 10.46883/ONC.2020.3410.0432

[39] Bonuomo V, Ferrarini I, Dell’Eva M, Sbisà E, Krampera M, Visco C. COVID-19 (SARS-CoV-2 infection) in lymphoma patients: a review. World J Virol. 2021;10:312–25. 34909405 10.5501/wjv.v10.i6.312 PMC8641038

[40] Visco C, Marcheselli L, Mina R, Sassone M, Guidetti A, Penna D, et al. A prognostic model for patients with lymphoma and COVID-19: a multicentre cohort study. Blood Adv. 2022;6:327–38. 34644385 10.1182/bloodadvances.2021005691 PMC8516438

[41] Harrington P, Doores KJ, Radia D, O’Reilly A, Lam HPJ, Seow J, et al. Single dose of BNT162b2 mRNA vaccine against severe acute respiratory syndrome coronavirus-2 (SARS-CoV-2) induces neutralising antibody and polyfunctional T-cell responses in patients with chronic myeloid leukaemia. Br J Haematol. 2021;194:999–1006. 34085278 10.1111/bjh.17568 PMC8239833

[42] Rea D, Mauro MJ, Cortes JE, Jiang Q, Pagnano KB, Ongondi M, et al. COVID-19 in patients (pts) with chronic myeloid leukemia (CML): results from the International CML foundation (iCMLf) CML and COVID-19 (CANDID) study. Blood. 2020;136:46–7.

[43] Breccia M, Abruzzese E, Bocchia M, Bonifacio M, Castagnetti F, Fava C, et al. Chronic myeloid leukemia management at the time of the COVID-19 pandemic in Italy a campus CML survey. Leukemia. 2020;34:2260–1. 32555369 10.1038/s41375-020-0904-z PMC7301058

[44] Neparidze N, Wang R, Zeidan AM, Podoltsev NA, Shallis RM, Ma X, et al. Changes in multiple myeloma treatment patterns during the early COVID-19 pandemic period. Leukemia. 2022;36:2136–9. 35761025 10.1038/s41375-022-01633-x PMC9243711

[45] Garnica M, Crusoe EDQ, Ribeiro G, Bittencourt R, Magalhães RJP, Zanella KR, et al. COVID-19 in multiple myeloma patients: frequencies and risk factors for hospitalization, ventilatory support, intensive care admission and mortality–cooperative registry from the Grupo Brasileiro de Mieloma Multiplo (GBRAM). Hematol Transfus Cell Ther. 2023;46:153–60. 37718131 10.1016/j.htct.2023.08.002 PMC11150487

[46] Chu Y, Liu Y, Fang X, Jiang Y, Ding M, Ge X, et al. The epidemiological patterns of non-Hodgkin lymphoma: global estimates of disease burden, risk factors, and temporal trends. Front Oncol. 2023;13:1059914. 37333805 10.3389/fonc.2023.1059914 PMC10272809

[47] Attal M, Lauwers-Cances V, Hulin C, Leleu X, Caillot D, Escoffre M, et al. Lenalidomide, bortezomib, and dexamethasone with transplantation for myeloma. N Engl J Med. 2017;376:1311–20. 28379796 10.1056/NEJMoa1611750 PMC6201242

[48] Li W, Wang D, Guo J, Yuan G, Yang Z, Gale RP, et al. COVID-19 in persons with chronic myeloid leukaemia. Leukemia. 2020;34:1799–804. 32424293 10.1038/s41375-020-0853-6 PMC7233329

[49] Vakiti A, Mewawalla P. Acute Myeloid Leukemia. StatPearls. Florida: StatPearls Publishing; 2024. 29939652

[50] Modemann F, Ghandili S, Schmiedel S, Weisel K, Bokemeyer C, Fiedler W. COVID-19 and adult acute leukemia: our knowledge in progress. Cancers. 2022;14:3711. 35954374 10.3390/cancers14153711 PMC9367547

[51] Aureli A, Marziani B, Venditti A, Sconocchia T, Sconocchia G. Acute lymphoblastic leukemia immunotherapy treatment: now, next, and beyond. Cancers (Basel). 2023;15:3346. 37444456 10.3390/cancers15133346 PMC10340788

[52] Cattaneo C, Daffini R, Pagani C, Salvetti M, Mancini V, Borlenghi E, et al. Clinical characteristics and risk factors for mortality in hematologic patients affected by COVID-19. Cancer. 2020;126:5069–76. 32910456 10.1002/cncr.33160

[53] Yigenoglu TN, Ata N, Altuntas F, Bascı S, Dal MS, Korkmaz S, et al. The outcome of COVID-19 in patients with hematological malignancy. J Med Virol. 2021;93:1099–104. 32776581 10.1002/jmv.26404 PMC7436524

[54] Galimberti S, Petrini M, Baratè C, Ricci F, Balducci S, Grassi S, et al. Tyrosine kinase inhibitors play an antiviral action in patients affected by chronic myeloid leukemia: a possible model supporting their use in the fight against SARS-CoV-2. Front Oncol. 2020. 10.3389/fonc.2020.01428. 33014780 10.3389/fonc.2020.01428 PMC7493657

[55] Sisk JM, Frieman MB, Machamer CE. Coronavirus S protein-induced fusion is blocked prior to hemifusion by ABL kinase inhibitors. J Gen Virol. 2018;99:619–30. 29557770 10.1099/jgv.0.001047 PMC6537626

[56] Coleman CM, Sisk JM, Mingo RM, Nelson EA, White JM, Frieman MB. Abelson kinase inhibitors are potent inhibitors of severe acute respiratory syndrome coronavirus and middle east respiratory syndrome coronavirus fusion. J Virol. 2016;90:8924–33. 27466418 10.1128/JVI.01429-16 PMC5021412

[57] Martínez-López J, Mateos MV, Encinas C, Sureda A, Hernández-Rivas J, Lopez de la Guía A, et al. Multiple myeloma and SARS-CoV-2 infection: clinical characteristics and prognostic factors of inpatient mortality. Blood Cancer J. 2020;10:103. 33077708 10.1038/s41408-020-00372-5 PMC7570395

[58] Chari A, Samur MK, Martinez-Lopez J, Cook G, Biran N, Yong K, et al. Clinical features associated with COVID-19 outcome in multiple myeloma: first results from the International Myeloma Society data set. Blood. 2020;136:3033–40. 33367546 10.1182/blood.2020008150 PMC7759145

[59] Shang Y, Wang W, Liang Y, Kaweme NM, Wang Q, Liu M, et al. Development of a risk assessment model for early grade ≥ 3 infection during the first 3 months in patients newly diagnosed with multiple myeloma based on a multicenter real-world analysis in China. Front Oncol. 2022;12: 772015. 35372017 10.3389/fonc.2022.772015 PMC8967980

[60] Susek KH, Gran C, Ljunggren H, Alici E, Nahi H. Outcome of COVID-19 in multiple myeloma patients in relation to treatment. Eur J Haematol. 2020;105:751–4. 32745304 10.1111/ejh.13502 PMC7436812

[61] Wang B, Van Oekelen O, Mouhieddine TH, Del Valle DM, Richter J, Cho HJ, et al. A tertiary center experience of multiple myeloma patients with COVID-19: lessons learned and the path forward. J Hematol Oncol. 2020;13:94. 32664919 10.1186/s13045-020-00934-x PMC7359431

[62] von Metzler I, Campe J, Huenecke S, Raab MS, Goldschmidt H, Schubert R, et al. COVID-19 in multiple-myeloma patients: cellular and humoral immunity against SARS-CoV-2 in a short- and long-term view. J Mol Med (Berl). 2022;100:463–70. 34657968 10.1007/s00109-021-02114-x PMC8520766

[63] Ibrahem HY, Aly DH, Warda AEA, Farahat RA, Youssef RM, Abdelhamid MH, et al. Efficacy of tocilizumab in management of COVID-19 patients admitted to intensive care units: a multicenter retrospective cohort study. Medicina. 2022;59:53. 36676678 10.3390/medicina59010053 PMC9864835

[64] Zheng R, Mieth K, Bennett C, Miller C, Anderson LD, Chen M, et al. Clinical features and risk stratification of multiple myeloma patients with COVID-19. Cancers (Basel). 2023;15:3598. 37509261 10.3390/cancers15143598 PMC10377341

[65] Krejci M, Pour L, Adam Z, Sandecka V, Stork M, Sevcikova S, et al. Outcome of COVID-19 infection in 50 multiple myeloma patients treated with novel drugs: single-center experience. Ann Hematol. 2021;100:2541–6. 34309714 10.1007/s00277-021-04594-w PMC8310901

[66] Cook G, John Ashcroft A, Pratt G, Popat R, Ramasamy K, Kaiser M, et al. Real-world assessment of the clinical impact of symptomatic infection with severe acute respiratory syndrome coronavirus (COVID-19 disease) in patients with multiple myeloma receiving systemic anti-cancer therapy. Br J Haematol. 2020. 10.1111/bjh.16874. 32438482 10.1111/bjh.16874 PMC7280609

[67] Ehsan H, Britt A, Voorhees PM, Paul B, Bhutani M, Varga C, et al. Retrospective review of outcomes of multiple myeloma (MM) patients with COVID-19 infection (two-center study). Clin Lymphoma Myeloma Leuk. 2023;23:273–8. 36797155 10.1016/j.clml.2023.01.006 PMC9847363

[68] Hultcrantz M, Richter J, Rosenbaum CA, Patel D, Smith EL, Korde N, et al. COVID-19 infections and clinical outcomes in patients with multiple myeloma in New York city: a cohort study from five academic centers. Blood Cancer Discov. 2020;1:234–43. 34651141 10.1158/2643-3230.BCD-20-0102 PMC7668224

[69] Tığlıoğlu P, Albayrak M, Tığlıoğlu M, Öztürk HBA, Aras MR, Sağlam B, et al. The outcome of COVID-19 in patients with hematological malignancy. Memo. 2022;15:83–9. 34904019 10.1007/s12254-021-00775-5 PMC8655323

[70] Oliva A, Curtolo A, Volpicelli L, Cancelli F, Borrazzo C, Cogliati Dezza F, et al. Clinical course of Coronavirus Disease-19 in patients with haematological malignancies is characterized by a longer time to respiratory deterioration compared to non-haematological ones: results from a case-control study. Infection. 2022;50:1373–82. 35781785 10.1007/s15010-022-01869-w PMC9251021

[71] Duléry R, Lamure S, Delord M, Di Blasi R, Chauchet A, Hueso T, et al. Prolonged in-hospital stay and higher mortality after COVID-19 among patients with non-Hodgkin lymphoma treated with B-cell depleting immunotherapy. Am J Hematol. 2021;96:934–44. 33909916 10.1002/ajh.26209 PMC8212109

[72] Pagano L, Salmanton-García J, Marchesi F, Busca A, Corradini P, Hoenigl M, et al. COVID-19 infection in adult patients with hematological malignancies: a European Hematology Association Survey (EPICOVIDEHA). J Hematol Oncol. 2021;14:168. 34649563 10.1186/s13045-021-01177-0 PMC8515781

[73] Marchesi F, Salmanton-García J, Emarah Z, Piukovics K, Nucci M, López-García A, et al. COVID-19 in adult acute myeloid leukemia patients: a long-term follow-up study from the european hematology Association survey (EPICOVIDEHA). Haematologica. 2022;108:22–33. 10.3324/haematol.2022.280847 PMC9827164 35545919

[74] Goldman JD, Robinson PC, Uldrick TS, Ljungman P. COVID-19 in immunocompromised populations: implications for prognosis and repurposing of immunotherapies. J Immunother Cancer. 2021;9: e002630. 34117116 10.1136/jitc-2021-002630 PMC8206176

[75] Grivas P, Khaki AR, Wise-Draper TM, French B, Hennessy C, Hsu C-Y, et al. Association of clinical factors and recent anticancer therapy with COVID-19 severity among patients with cancer: a report from the COVID-19 and cancer consortium. Ann Oncol. 2021;32:787–800. 33746047 10.1016/j.annonc.2021.02.024 PMC7972830

[76] Chavez-MacGregor M, Lei X, Zhao H, Scheet P, Giordano SH. Evaluation of COVID-19 mortality and adverse outcomes in us patients with or without cancer. JAMA Oncol. 2022;8:69–78. 34709356 10.1001/jamaoncol.2021.5148 PMC8554684

[77] Várnai C, Palles C, Arnold R, Curley HM, Purshouse K, Cheng VWT, et al. Mortality among adults with cancer undergoing chemotherapy or immunotherapy and infected with COVID-19. JAMA Netw Open. 2022;5: e220130. 35188551 10.1001/jamanetworkopen.2022.0130 PMC8861846

[78] Sharafeldin N, Bates B, Song Q, Madhira V, Yan Y, Dong S, et al. Outcomes of COVID-19 in patients with cancer: report from the national COVID cohort collaborative (N3C). J Clin Oncol. 2021;39:2232–46. 34085538 10.1200/JCO.21.01074 PMC8260918

[79] Lee CY, Shah MK, Hoyos D, Solovyov A, Douglas M, Taur Y, et al. Prolonged SARS-CoV-2 infection in patients with lymphoid malignancies. Cancer Discov. 2022;12:62–73. 34753749 10.1158/2159-8290.CD-21-1033 PMC8758535

[80] Cheng GS, Evans SE. The paradox of immunosuppressants and COVID-19. Eur Respir J. 2022;59:2102828. 34795039 10.1183/13993003.02828-2021 PMC8607867

[81] Ripa M, Galli L, Poli A, Oltolini C, Spagnuolo V, Mastrangelo A, et al. Secondary infections in patients hospitalized with COVID-19: incidence and predictive factors. Clin Microbiol Infect. 2021;27:451–7. 33223114 10.1016/j.cmi.2020.10.021 PMC7584496

[82] Zappasodi P, Cattaneo C, Valeria Ferretti V, Mina R, José María Ferreri A, Merli F, et al. Secondary infections worsen the outcome of COVID-19 in patients with hematological malignancies: a report from the ITA-HEMA-COV. Hematol Oncol. 2022;40:846–56. 35854643 10.1002/hon.3048 PMC9349965

[83] Casetti IC, Borsani O, Rumi E. COVID-19 in patients with hematologic diseases. Biomedicines. 2022;10:3069. 36551825 10.3390/biomedicines10123069 PMC9775038

[84] Pinato DJ, Tabernero J, Bower M, Scotti L, Patel M, Colomba E, et al. Prevalence and impact of COVID-19 sequelae on treatment and survival of patients with cancer who recovered from SARS-CoV-2 infection: evidence from the on covid retrospective, multicentre registry study. Lancet Oncol. 2021;22:1669–80. 34741822 10.1016/S1470-2045(21)00573-8 PMC8565932

[85] Chen S, Guan F, Candotti F, Benlagha K, Camara NOS, Herrada AA, et al. The role of B cells in COVID-19 infection and vaccination. Front Immunol. 2022;13:988536. 36110861 10.3389/fimmu.2022.988536 PMC9468879

[86] Shah N, Mustafa SS, Vinh DC. Management of secondary immunodeficiency in hematological malignancies in the era of modern oncology. Crit Rev Oncol Hematol. 2023;181: 103896. 36528276 10.1016/j.critrevonc.2022.103896

[87] Barahona-Correa JE, Rueda-Ortiz C, López M-J, Gualtero S, Arevalo-Zambrano M. COVID-19 infection during blinatumomab therapy: is safety a dilemma? SAGE Open Med Case Rep. 2023;11:2050313X221148548. 36643709 10.1177/2050313X221148548 PMC9834623

[88] Cesaro S, Ljungman P, Mikulska M, Hirsch HH, von Lilienfeld-Toal M, Cordonnier C, et al. Recommendations for the management of COVID-19 in patients with haematological malignancies or haematopoietic cell transplantation, from the 2021 European Conference on Infections in Leukaemia (ECIL 9). Leukemia. 2022;36:1467. 35488021 10.1038/s41375-022-01578-1 PMC9053562

[89] Salmanton-García J, Marchesi F, Farina F, Weinbergerová B, Itri F, Dávila-Valls J, et al. Decoding the historical tale: COVID-19 impact on haematological malignancy patients—EPICOVIDEHA insights from 2020 to 2022. E Clin Med. 2024;71:102553. 10.1016/j.eclinm.2024.102553 PMC10963230 38533127

[90] Cesaro S, Mikulska M, Hirsch HH, Styczynski J, Meylan S, Cordonnier C, et al. Update of recommendations for the management of COVID-19 in patients with haematological malignancies, haematopoietic cell transplantation and CAR T therapy, from the 2022 European Conference on Infections in Leukaemia (ECIL 9). Leukemia. 2023;37:1933. 37460673 10.1038/s41375-023-01938-5 PMC10457191

[91] Perry C, Luttwak E, Balaban R, Shefer G, Morales MM, Aharon A, et al. Efficacy of the BNT162b2 mRNA COVID-19 vaccine in patients with B-cell non-Hodgkin lymphoma. Blood Adv. 2021;5:3053–61. 34387648 10.1182/bloodadvances.2021005094 PMC8362658

[92] Blennow O, Salmanton-García J, Nowak P, Itri F, Van Doesum J, López-García A, et al. Outcome of infection with omicron SARS-CoV-2 variant in patients with hematological malignancies: an EPICOVIDEHA survey report. Am J Hematol. 2022;97:E312–7. 35702878 10.1002/ajh.26626 PMC9349555

[93] Del Poeta G, Bomben R, Polesel J, Rossi FM, Pozzo F, Zaina E, et al. COVID-19 vaccination: evaluation of risk for protection failure in chronic lymphocytic leukemia patients. Hematol Oncol. 2021;39:712–4. 34462939 10.1002/hon.2916 PMC8652757

[94] Corti C, Antonarelli G, Scotté F, Spano JP, Barrière J, Michot JM, et al. Seroconversion rate after vaccination against COVID-19 in patients with cancer-a systematic review. Ann Oncol. 2022;33:158–68. 34718117 10.1016/j.annonc.2021.10.014 PMC8552625

[95] Thakkar A, Gonzalez-Lugo JD, Goradia N, Gali R, Shapiro LC, Pradhan K, et al. Seroconversion rates following COVID-19 vaccination among patients with cancer. Cancer Cell. 2021;39:1081-1090.e2. 34133951 10.1016/j.ccell.2021.06.002 PMC8179248

[96] Morawska M. Reasons and consequences of COVID-19 vaccine failure in patients with chronic lymphocytic leukemia. Eur J Haematol. 2022;108:91–8. 34717004 10.1111/ejh.13722 PMC8652891

[97] Aleman A, Upadhyaya B, Tuballes K, Kappes K, Gleason CR, Beach K, et al. Variable cellular responses to SARS-CoV-2 in fully vaccinated patients with multiple myeloma. Cancer Cell. 2021;39:1442–4. 34706273 10.1016/j.ccell.2021.09.015 PMC8523488

[98] Oekelen OV, Gleason CR, Agte S, Srivastava K, Beach KF, Aleman A, et al. Highly variable SARS-CoV-2 spike antibody responses to two doses of COVID-19 RNA vaccination in patients with multiple myeloma. Cancer Cell. 2021;39:1028–30. 34242572 10.1016/j.ccell.2021.06.014 PMC8238657

[99] Wiedmeier-Nutor JE, Iqbal M, Rosenthal AC, Bezerra ED, Garcia-Robledo JE, Bansal R, et al. Response to COVID-19 vaccination post-CAR T therapy in patients with non-Hodgkin lymphoma and multiple myeloma. Clin Lymphoma Myeloma Leuk. 2023;23:456–62. 37003846 10.1016/j.clml.2023.03.002 PMC9990888

[100] Zhang T, Tian W, Wei S, Lu X, An J, He S, et al. Multidisciplinary recommendations for the management of CAR-T recipients in the post-COVID-19 pandemic era. Exp Hematol Oncol. 2023;12:66. 37501090 10.1186/s40164-023-00426-x PMC10375673

[101] Giesen N, Busch E, Schalk E, Beutel G, Rüthrich MM, Hentrich M, et al. AGIHO guideline on evidence-based management of COVID-19 in cancer patients: 2022 update on vaccination, pharmacological prophylaxis and therapy in light of the omicron variants. Eur J Cancer. 2023;181:102–18. 36652889 10.1016/j.ejca.2022.11.030 PMC9737523

[102] Owen C, Robinson S, Christofides A, Sehn LH. A canadian perspective: monoclonal antibodies for pre- and post-exposure protection from COVID-19 in vulnerable patients with hematological malignancies. Curr Oncol. 2022;29:3940–9. 35735424 10.3390/curroncol29060315 PMC9222187

[103] Levin MJ, Ustianowski A, De Wit S, Launay O, Avila M, Templeton A, et al. Intramuscular AZD7442 (Tixagevimab-Cilgavimab) for prevention of COVID-19. N Engl J Med. 2022;386:2188–200. 35443106 10.1056/NEJMoa2116620 PMC9069994

[104] O’Brien MP, Forleo-Neto E, Musser BJ, Isa F, Chan KC, Sarkar N, et al. Subcutaneous REGEN-COV antibody combination to prevent COVID-19. N Engl J Med. 2021;385:1184–95. 34347950 10.1056/NEJMoa2109682 PMC8362593

[105] Stuver R, Shah GL, Korde NS, Roeker LE, Mato AR, Batlevi CL, et al. Activity of AZD7442 (tixagevimab-cilgavimab) against Omicron SARS-CoV-2 in patients with hematologic malignancies. Cancer Cell. 2022;40:590–1. 35598602 10.1016/j.ccell.2022.05.007 PMC9108069

[106] Najjar-Debbiny R, Gronich N, Weber G, Khoury J, Amar M, Stein N, et al. Effectiveness of paxlovid in reducing severe coronavirus disease 2019 and mortality in high-risk patients. Clin Infect Dis. 2022;76:e342–9. 10.1093/cid/ciac443 PMC9214014 35653428

[107] Gottlieb RL, Vaca CE, Paredes R, Mera J, Webb BJ, Perez G, et al. Early remdesivir to prevent progression to severe COVID-19 in outpatients. N Engl J Med. 2022;386:305–15. 34937145 10.1056/NEJMoa2116846 PMC8757570

[108] Hammond J, Leister-Tebbe H, Gardner A, Abreu P, Bao W, Wisemandle W, et al. oral nirmatrelvir for high-risk, nonhospitalized adults with COVID-19. N Engl J Med. 2022;386:1397–408. 35172054 10.1056/NEJMoa2118542 PMC8908851

[109] Jayk Bernal A, Gomes da Silva MM, Musungaie DB, Kovalchuk E, Gonzalez A, Delos Reyes V, et al. Molnupiravir for oral treatment of COVID-19 in nonhospitalized patients. N Engl J Med. 2022;386:509–20. 34914868 10.1056/NEJMoa2116044 PMC8693688

[110] Giesen N, Sprute R, Rüthrich M, Khodamoradi Y, Mellinghoff SC, Beutel G, et al. Evidence-based management of COVID-19 in cancer patients: guideline by the Infectious Diseases Working Party (AGIHO) of the German Society for Haematology and Medical Oncology (DGHO). Eur J Cancer. 2020;140:86–104. 33068941 10.1016/j.ejca.2020.09.009 PMC7505554

